# Analysis of global gene expression changes in human bronchial epithelial cells exposed to spores of the allergenic fungus, *Alternaria alternata*

**DOI:** 10.3389/fmicb.2013.00196

**Published:** 2013-07-19

**Authors:** M. C. Babiceanu, B. A. Howard, A. C. Rumore, H. Kita, C. B. Lawrence

**Affiliations:** ^1^Virginia Bioinformatics Institute, Virginia TechBlacksburg, VA, USA; ^2^Department of Biological Sciences, Virginia TechBlacksburg, VA, USA; ^3^Department of Immunology, Mayo ClinicRochester, MN, USA

**Keywords:** Alternaria, epithelial cells, allergy and immunology, gene expression, immunology

## Abstract

Exposure and sensitivity to ubiquitous airborne fungi such as *Alternaria alternata* have long been implicated in the development, onset, and exacerbation of chronic allergic airway disorders. This present study is the first to investigate global changes in host gene expression during the interaction of cultured human bronchial epithelial cells and live Alternaria spores. In *in vitro* experiments human bronchial epithelial cells (BEAS-2B) were exposed to spores or media alone for 24 h. RNA was collected from three biological replicates per treatment and was used to assess changes in gene expression patterns using Affymetrix Human Genome U133 Plus 2.0 Arrays. In cells treated with Alternaria spores compared to controls, 613 probe sets representing 460 individual genes were found differentially expressed (*p* ≤ 0.05). In this set of 460 statistically significant, differentially expressed genes, 397 genes were found to be up-regulated and 63 were down-regulated. Of these 397 up-regulated genes, 156 genes were found to be up-regulated ≥2 fold. Interestingly, none of the 63 down-regulated genes were found differentially expressed at ≤−2 fold. Differentially expressed genes were identified following statistical analysis and subsequently used for pathway and network evaluation. Interestingly, many cytokine and chemokine immune response genes were up-regulated with a particular emphasis on interferon-inducible genes. Genes involved in cell death, retinoic acid signaling, and TLR3 response pathways were also significantly up-regulated. Many of the differentially up-regulated genes have been shown in other systems to be associated with innate immunity, inflammation and/or allergic airway diseases. This study now provides substantial information for further investigating specific genes and innate immune system pathways activated by Alternaria in the context of allergic airway diseases.

## Introduction

The airway epithelium has long been considered a physiochemical barrier characterized by having tight junctions or producing proteins and/or enzymes that collectively constitute a protective environment. Respiratory epithelial cells play an active role in innate immunity via recognition and response to pathogen associated molecular patterns (PAMPs) and/or specific pathogen-secreted immunogenic proteins. This innate immune response often includes the production of a variety of cytokines, chemokines, extracellular matrix and growth factors that may function to recruit other effector cells such as macrophages, neutrophils, eosinophils, and lymphocytes and ultimately shape the development of adaptive immune responses associated with complex allergic diseases like allergic or atopic asthma (Mills et al., 1999; Shin and Lee, 2010).

Human airways are continuously exposed to ubiquitous environmental fungal spores and hyphae. This exposure often occurs at a higher level and for a longer duration than that of pollen or other types of airborne allergen sources. The most common airborne fungi include species found within the genera Cladosporium, Alternaria, Penicillium, and Aspergillus, although a multitude of other species are commonly found in any type of environment and climate (Al-Doory and Domson, 1984; Bowyer and Denning, 2007).

Alternaria species are considered some of the most important fungi responsible for allergic reactions in humans (Al-Doory and Domson, 1984). Specifically, *A. alternata* is one of the most frequently found species as a causative agent of type I, IgE-mediated, hypersensitivity in indoor and outdoor environments, especially in regions with a warm and/or arid climate (Bowyer and Denning, 2007). It has been known for decades that sensitivity to *A*. *alternata* is a common cause of atopic or allergic asthma (Halonen et al., 1997). It has also been demonstrated that allergic rhinitis and upper respiratory tract symptoms can develop or become exacerbated when the level of Alternaria spores in the environment is high (Andersson et al., 2003). One study has shown that within the United States, 3.6% of the population is sensitized to mold in general and *A. alternata* in particular (Gergen and Turkeltaub, 1992).

Some of the immunological research regarding Alternaria-human interactions has centered upon the role of proteases found in commercially available antigen/allergen extracts or culture filtrates in regards to innate immune responses. In a recent paper it was reported that the cysteine protease activities in Alternaria extracts induced TSLP (thymic stromal lymphopoietin) in bronchial epithelial cells (BEAS-2B) in a protease-activated receptor 2 (PAR-2) dependent manner (Kouzaki et al., 2009). In a separate study, serine proteases found in *A. alternata* culture filtrate applied to human bronchial epithelial cells (16HBE14o-) induced changes in intracellular Ca^2+^ concentration and was associated with PAR-2 activation (Boitano et al., 2011). In another study, Alternaria extract induced eosinophil degranulation and was dependent on protease activity and PAR-2 (Matsuwaki et al., 2009). Besides these protease-centered studies, it was reported that Alternaria extracts induced patient nasal epithelial cells to express TLR2, TLR3, and TLR4 mRNAs much stronger than in the controls (Shin and Lee, 2010).

In a recent study (Oguma et al., 2011) microarray analysis was performed on the pulmonary NCIH292 cell line treated with culture extracts of Alternaria, Aspergillus, and Penicillium; however this microarray analysis focused on identifying genes, namely mucin-encoding genes, exclusively induced by *A. fumigatus*. Moreover, this study used fungal extracts and not living fungal spores or hyphae. In our present study, a microarray analysis approach was used for the first time to profile changes in gene expression in the human bronchial epithelial cell line BEAS-2B (Ke et al., 1988) exposed to live *A. alternata* spores, not extracts. We utilized spores from a strain of *A. alternata* (ATCC 6981) considered to be a robust allergenic type-strain for this species (Burge et al., 1989) and for which we have a recently sequenced genome (Lawrence et al., unpublished). The up-regulation of several pathways previously linked to allergic inflammation and related diseases were identified using Ingenuity Pathway Analysis (IPA) software. These pathways were found to be centered upon various genes including interferons, TLR-3, and retinoic acid, but have yet to be reported in the context of Alternaria-associated airway disorders.

## Results

### Analysis of differential gene expression using microarray

**Figure 1:**
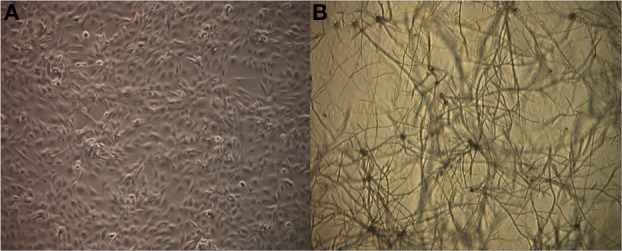
**Germinating *Alternaria alternata* spores in the presence of human airway epithelial cells.** BEAS-2B cells were incubated with live *A. alternata* spores for 24 h under normal conditions at 37°C, 5% CO_2_. Cultures were imaged using an inverted phase-contrast microscope. Magnification: 40×. **(A)** Untreated BEAS-2B cells, **(B)** BEAS-2B cells treated with *A. alternata* spores at 24 h.

In order to investigate global gene expression changes in airway epithelial cells in response to Alternaria we performed a comparative microarray analysis of a human bronchial epithelial cell line (BEAS-2B) stimulated with live spores of *A. alternata* with untreated (media only) cells serving as the control. The spores were allowed to germinate for 24 h during which no significant damage or morphological changes were observed in the epithelial cell (Figure 1). Spores readily germinated typically within 1 h at 37°C and grew slowly over the 24-h period of incubation. Cytotoxicity was overall quite low with only ~1.5% of cell death resulting from fungal spore treatment as measured by the LDH assay (data not shown). RNA was then extracted from three biological replicates of each sample type (spore treated or control cells) and transcriptional profiling experiments were completed using the Affymetrix Human Genome U133 Plus 2.0 Arrays.

After performing statistical analyses employing the Robust Multi-chip Average method (RMA) (Irizarry et al., 2003) using the R software package version 2.12 (http://www.r-project.org/), a statistically significant set of differentially expressed genes was identified. In cells treated with Alternaria spores compared to controls, 613 probe sets representing 460 individual genes were found differentially expressed (*p* ≤ 0.05). In this set of 460 statistically significant, differentially expressed genes, 397 genes were found to be up-regulated and 63 were down-regulated. Of these 397 up-regulated genes, 156 genes were found to be up-regulated ≥2 fold. Interestingly, none of the 63 down-regulated genes were found to be differentially expressed at ≤−2 fold. A subset of the top 30 genes of interest differentially expressed ≥2 fold are shown in Table 1. Many genes annotated as immunological response genes including those related to interferon signaling, chemokines, cytokines, and retinoic acid signaling, were evident in this data set. Interestingly, we did not find any evidence of up-regulation of interferon genes themselves (IFN-α, -β, -γ) at 24 h even though we found dramatic up-regulation of interferon response-associated genes.

**Table 1:** **Top 30 genes modulated in bronchial epithelial cells in response to exposure to *Alternaria alternata* spores**.

**Rank**	**Gene symbol**	**Fold change**	**Description**	**Genbank ID**	**Gene ontology biological process**
1	MXl	66.2	Myxovirus (influenza virus) resistance 1, interferon-inducible protein p78 (mouse)	NM_002462	Induction of apoptosis, defense response, signal transduction, response to virus, cytokine-mediated signaling pathway
2	IFI44L	64.1	Interferon-induced protein 44-like	NM_006820	Immune response
3	IFI44	39.2	Interferon-induced protein 44	BE049439	Response to virus
4	XAFl	31.2	XIAP associated factor 1	AI859280	Apoptosis, cytokine-mediated signaling pathway, type 1 interferon-mediated signaling pathway
5	OAS2	30.8	2′-5′-oligoadenylate synthetase 2, 69/71 kDa	AI651594	Immune response, cytokine-mediated signaling pathway, interferon-gamma-mediated signaling pathway
6	IFI27	25.2	Interferon, alpha-inducible protein 27	NM_005532	Induction of apoptosis by extracellular signals, activation of pro-apoptotic gene products, cytokine-mediated signaling pathway, type 1 interferon-mediated signaling pathway
7	IFIH1	19.3	Interferon induced with helicase C domain 1	AL080107	Negative regulation of type 1 interferon production, positive regulation of interferon-alpha production, positive regulation of interferon-beta production
8	CMPK2	18.7	Cytidine monophosphate (UMP-CMP) kinase 2, mitochondrial	AI742057	Pyrimidine nucleotide biosynthetic process, dTDP biosynthetic process, cellular response to lipopolysaccharide
9	MX2	17.9	Myxovirus (influenza virus) resistance 2	NM_002463	Defense response, cytokine-mediated signaling pathway, type 1 interferon-mediated signaling pathway
10	IFIT1	16.4	Interferon-induced protein with tetratricopeptide repeats 1	NM_001548	Intracellular transport of viral proteins in host cell, cytokme-mediated signaling pathway, negative regulation of protein binding, positive regulation of viral genome replication
11	HERC6	15	Hect domain and RLD 6	NM_017912	Protein modification process, protein ubiquitination involved in ubiquitin-dependent protein catabolic process
12	HERC5	13.9	Hect domain and RLD 5	NM_016323	Regulation of cyclin-dependent protein kinase activity, protein modification process, response to virus, ISGl5-protein conjugation, negative regulation of type 1 interferon production
13	IFIT3	13.7	Interferon-induced protein with tetratricopeptide repeats 3	NM_001549	Cytokine-mediated signaling pathway, cellular response to interferon-alpha, type 1 interferon-mediated signaling pathway
14	IFIT2	13.6	Interferon-induced protein with tetratricopeptide repeats 2	BE888744	Response to virus, cytokine-mediated signaling pathway, negative regulation of protein binding, cellular response to interferon-alpha
15	CDC2O	13.5	Cell division cycle 20 homolog (*S. cerevisiae*)	NM_001255	Cell cycle checkpoint, M phase of mitotic cell cycle, mitotic prometaphase
16	OAS1	13.2	2′,5′-oligoadenylate synthetase 1, 40/46kDa	NM_002534	Immune response, cytokine-mediated signaling pathway, interferon-gamma-mediated signaling pathway, type 1 interferon-mediated signaling pathway
17	TNFSF10	13.2	Tumor necrosis factor (ligand) superfamily, member 10	U57O59	Apoptosis, induction of apoptosis, activation of caspase activity, immune response
18	DDX58	12.1	DEAD (Asp-Glu-Ala-Asp) box polypeptide 58	AI304317	Defense response to virus by host, detection of virus, negative regulation of type 1 interferon production, positive regulation of interferon-beta production
19	RARRES1	12.1	Retinoic acid receptor responder (tazarotene induced) 1	NM_002888	Negative regulation of cell proliferation
20	ISG15	12	ISG15 ubiquitin-like modifier	NM_005101	Cell–cell signaling, response to virus, cytokine-mediated signaling pathway, modification-dependent protein catabolic process, ISGl5-protein conjugation
21	USP18	11.9	Ubiquitin specific peptidase 18	NM_017414	Cytokine-mediated signaling pathway, type 1 interferon-mediated signaling pathway, regulation of type 1 interferon-mediated signaling pathway
22	RTP4	10	Receptor (chemosensory) transporter protein 4	NM_022147	Protein targeting to membrane
23	STAT1	9.8	Signal transducer and activator of transcription 1, 9lkDa	NM_007315	Regulation of transcription, transcription from RNA polymerase II promoter, induction of apoptosis, activation of caspase activity, signal transduction, JAK-STAT cascade, tyrosine phosphorylation of STAT protein
24	OASL	8.5	2′-5′-oligoadenylate synthetase-like	NM_003733	Immune response, interferon-gamma-mediated signaling pathway, type 1 interferon-mediated signaling pathway
25	DDX6O	8	DEAD (Asp-Glu-Ala-Asp) box polypeptide 60	NM_017631	**–**
26	IFITM1	7.8	Interferon induced transmembrane protein 1 (9-27)	NM_003641	Cell surface receptor linked signaling pathway, negative regulation of cell proliferation, response to virus, cytokine-mediated signaling pathway
27	OAS3	7.7	2′-5′-oligoadenylate synthetase 3, lOOkDa	R13458	Immune response, cytokine-mediated signaling pathway, interferon-gamma-mediated signaling pathway, type 1 interferon-mediated signaling pathway
28	DHRS3	7.3	Dehydrogenase/reductase (SDR family) member 3	NM_004753	Retinol metabolic process
29	PARP9	7.2	Poly (ADP-ribose) polymerase family, member 9	AI738416	Cell migration
30	IFI6	7	Interferon, alpha-inducible protein 6	NM_022873	Immune response, cytokine-mediated signaling pathway, negative regulation of caspase activity

The top 30 differentially expressed (up-regulated) genes are shown along with the gene description, the relative fold change of the target genes, and the NCBI GenBank ID. A description of Gene Ontology (GO) annotation of the biological processes is also depicted.

### Analysis of pathways and gene networks using ingenuity pathway system (IPA)

**Figure 2:**
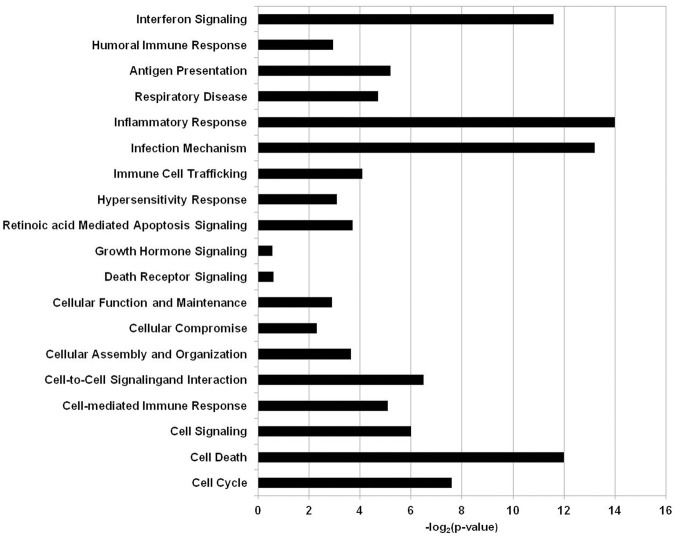
**Biological functions and processes in human airway cells exposed to *Alternaria alternata* spores.** The x-axis depicts the biological processes and y-axis is the −log (*p*-value) as produced by IPA analysis.

All of the statistically significant output data that was obtained from the RMA statistical analysis regardless of fold change was further analyzed using a pathway and network analysis system Ingenuity Pathway System (IPA, Ingenuity Systems, http://www.ingenuity.com). The clusters of biological processes corresponding to genes that were differentially expressed when the BEAS-2B human epithelial cell line was exposed to *A. alternata* were generated with IPA software and are visualized in Figure 2. The biological processes corresponding to the gene sets were shown to be involved to a large extent in immunological processes, cell growth, cell signaling, or cell death/apoptosis.

The first cluster we examined was the immunological group and includes genes that pertain to C-X-C motif like proteins: CXCL1 (Chemokine ligand 1), CXCL6, (Chemokine ligand 6), CXCL10 (Chemokine ligand 10), CXCL11 (Chemokine ligand 11), and a gene that is also a chemokine but from the C-C motif group CCL2 (chemokine ligand 2). In the same group we also unveiled receptors like the CD (cluster of differentiation) containing proteins like TLR3 (Toll-like receptor 3 also known as CD283), ICAM (Inter-Cellular Adhesion Molecule 1 also known as CD54), CD14, CD47, JAG1 (also designated as CD339**),** IL-7R (Interleukin-7 receptor), and MYD88 (Myeloid differentiation primary response gene 88). Under the same immunological umbrella, another group was identified and contained interleukins like IL1A (Interleukin-1 alpha), IL8 (Interleukin-8), and IL32 (Interleukin-32). Another cluster contained CEBPD (CCAAT/enhancer-binding protein, delta), TNFRSF21 (Tumor necrosis factor receptor superfamily, member 21), TNFSF15 (Tumor necrosis factor superfamily 15), TNFSF13B (Tumor necrosis factor superfamily 13B), STAT1 and STAT2 (Signal transducer and activator of transcription 1,2) and MUC1 (cell surface associated Mucin 1).

**Figure 3:**
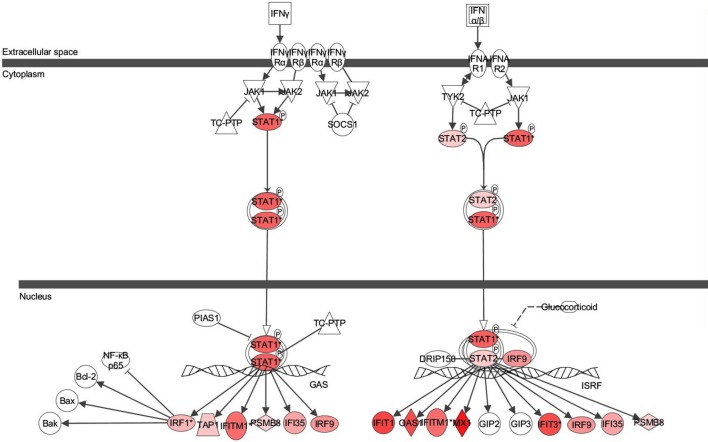
**Ingenuity pathway analysis (IPA) of Interferon Signaling Pathway of genes differentially expressed in the bronchial epithelial cells exposed to *Alternaria alternata* spores.** Genes that are significantly up-regulated are shown in red. The intensity of red corresponds to an increase in fold change levels of spore treated cells compared to control. Genes in white did not exhibit significant changes in expression at 24 h post inoculation with Alternaria. The network was generated with IPA (Ingenuity Pathways System).

**Figure 4:**
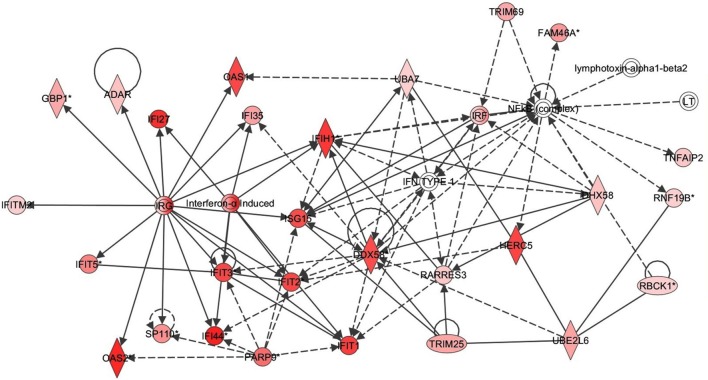
**Interferon response gene network in respiratory epithelial cells exposed to *Alternaria alternata* spores.** Genes that are significantly up-regulated are shown in red. The intensity of red corresponds to an increase in fold change levels of spore treated cells compared to control. Genes in white did not exhibit significant changes in expression at 24 h post inoculation with Alternaria. The network was generated with IPA (Ingenuity Pathways System).

**Figure 5:**
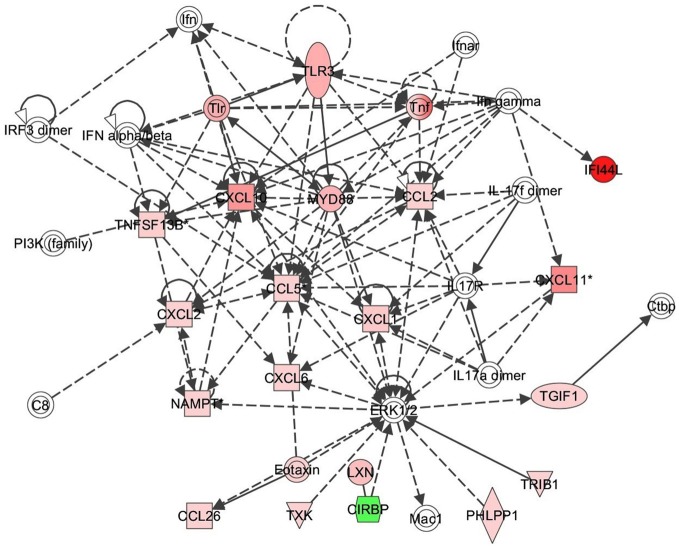
**TLR3 response signaling inflammatory network in response to *Alternaria alternata* spores.** Genes that are significantly up-regulated are shown in red and genes that are down-regulated are shown in green. The intensity of red or green corresponds to an increase or decrease in fold change levels of spore treated cells compared to control. Genes in white did not exhibit significant changes in expression at 24 h post inoculation with Alternaria. The network was generated with IPA (Ingenuity Pathways System).

**Figure 6:**
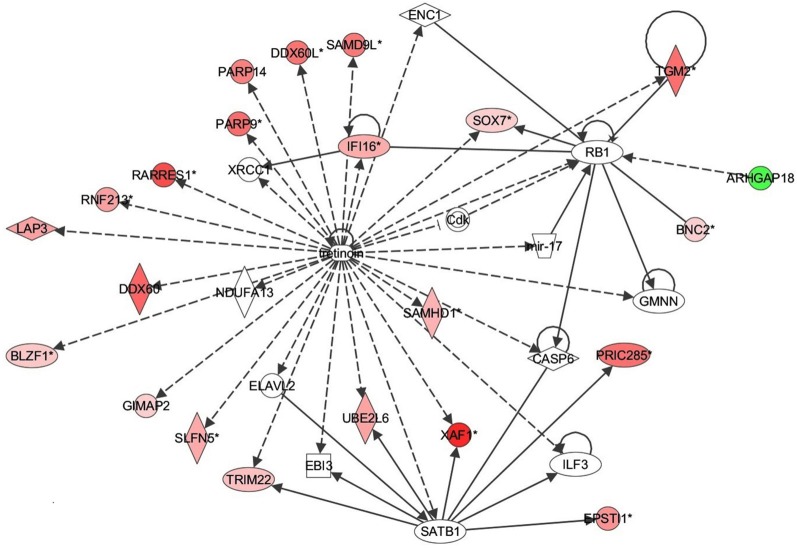
**Retinoic acid response signaling inflammatory mechanisms in bronchial epithelial cells exposed to *Alternaria alternata* spores.** Genes that are significantly up-regulated are shown in red and genes that are down-regulated are shown in green. The intensity of red or green corresponds to an increase or decrease in fold change levels of spore treated cells compared to control. Genes in white did not exhibit significant changes in expression at 24 h post inoculation with Alternaria. The network was generated with IPA (Ingenuity Pathways System).

Next the expression of different pathways was analyzed with IPA and several gene interaction networks were created. The differentially expressed genes due to fungal spore exposure appear in either red or green and up-regulated or down-regulated, respectively (Figures 3–6). We focused our analyses primarily upon pathways and sets of genes with a previous association with allergic inflammation including interferons, other innate immunity-associated genes, and pathways including Toll-like receptors and retinoic acid signaling. For example, differentially expressed genes were mapped onto several pathways including the interferon pathway (Figures 3, 4). A network was also created to illustrate TLR3 connections with many chemokines, chemokine receptors, and interferon inducible genes (Figure 5). Finally, a network centered upon retinoic acid was created linking the response to this metabolite with interferon signaling (Figure 6).

### Validation of differential gene expression with qRT-PCR and ELISA

**Figure 7:**
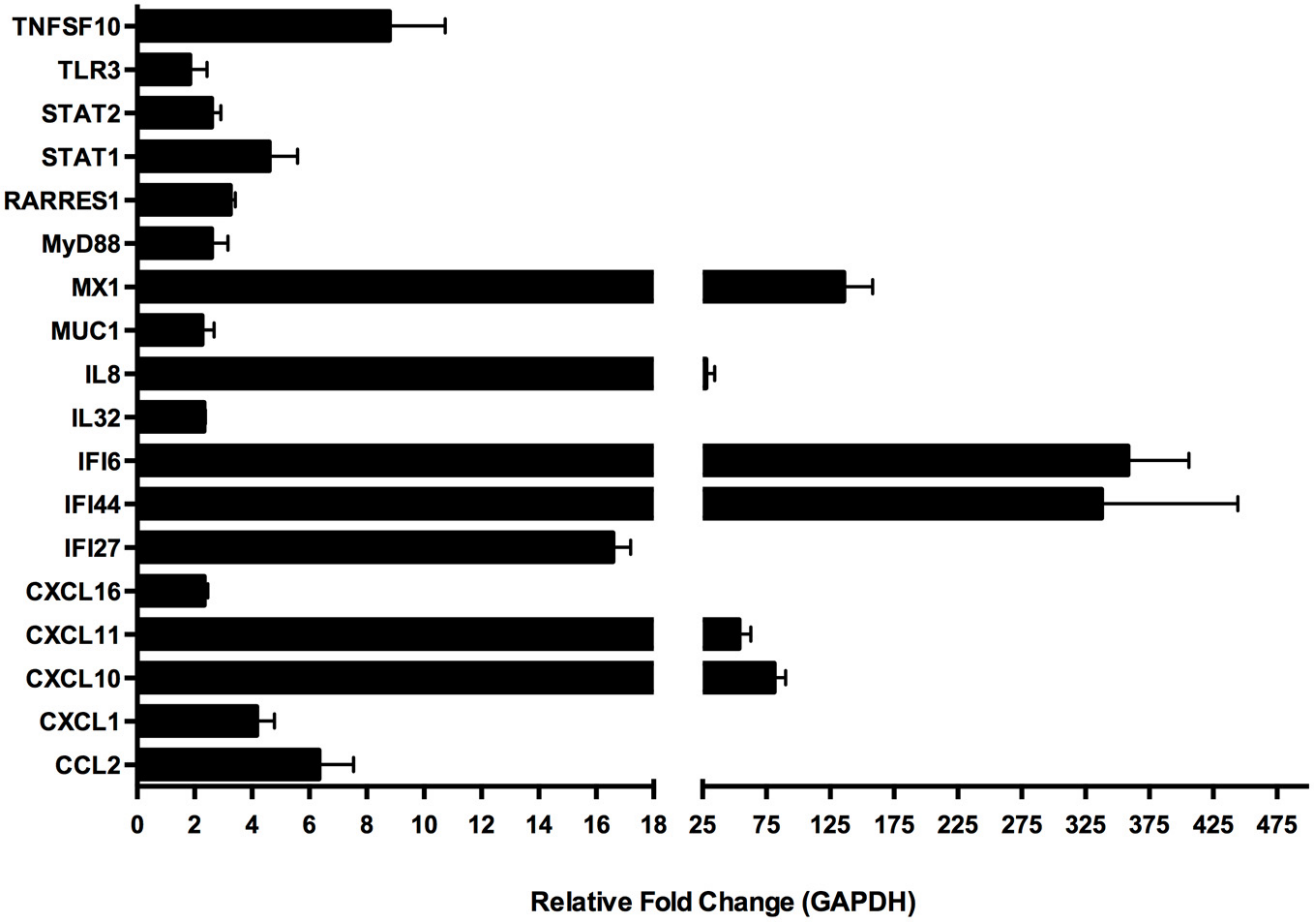
**Quantitative real-time (qRT)-PCR amplification of select genes in human bronchial epithelial cells exposed to *Alternaria alternata* spores.** Genes were selected from the larger microarray dataset based on known immunological function and differential expression levels between control and spore-treated cells. Differential gene expression was confirmed by qRT-PCR using RNA samples collected from an independent experiment.

Several gene targets were selected to validate the microarray data via quantitative real-time PCR (qRT-PCR) from mRNA prepared from a separate experiment conducted under identical culture conditions as used for the microarray experiment RNA preparation (Figure 7). We were able to confirm our microarray data for this subset of genes. The genes were selected based on their known function, especially in regards to immunological relevance. These genes were: CCL2, CXCL1, CXCL10, CXCL11, CXCL16, Il32, IL8, MUC1, STAT2, IFI27, IFI44, IFI6, MX1, MyD88, RARRES1, STAT1 and TLR3 and the primers that were used for amplification of the transcripts corresponding to the selected genes are found in Table 2. Among the genes with known immunological function, interferon inducible genes 6 (IFI6) and 44 (IFI44) were strongly induced in BEAS-2B cells upon treatment with *A. alternata* spores (358- and 326-fold up-regulation vs. control, respectively). The interferon-induced GTP-binding protein, MX1, was also highly expressed in treated cells (136-fold up-regulation). The C-X-C motif chemokines 10 (CXCL10) and 11 (CXCL11) were up-regulated 81- and 54-fold respectively, and interleukin-8 underwent 28-fold induction. Lower-level but significant up-regulation was observed for a variety of genes, including: IFI27 (17-fold), TNFSF10 (9-fold), the chemokine CCL2 (6-fold), and transcription factors STAT1 (4.6-fold) and STAT2 (2.6-fold).

**Table 2:** **Primer sequences used for qRT-PCR based validation of microarray results**.

**Gene**	**Forward primer**	**Reverse primer**
GAPDH	CTCTGACTTCAACAGCGAC	TGGTCCAGGGGTCTTACT
CCL2	CCTCCAGCATGAAAGTCTCTG	TCTGCACTGAGATCTTCCTATTG
CXCL1	AAGTGTGAACGTGAAGTCCC	GTCACTGTTCAGCATCTTTTCG
CXCL10	GCATCAGCATTAGTAATCAACCTG	TGGCCTTCGATTCTGGATTC
CXCL11	GTGTGAAGGGCATGGCTATAG	GCCACTTTCACTGCTTTTACC
CXCL16	GCTGAGAGTAAACCCCAAAAC	TCCAGACAAACTTGCTTCCC
IFI27	TTGACCAAGTTCATCCTGGG	CCCTGGCATGGTTCTCTTC
IFI44	AGTATGGCAGTGACAACTCG	GTCAAGCAAAACTCCATTACGG
IFI6	CTGGTCTGCGATCCTGAATG	CCATCAGGGCACCAATATTACC
Il32	TGCACCAGGCCATAGAAAG	GGTAGCCCTCTTTGAAGTCG
IL8	TCCTGATTTCTGCAGCTCTG	GTCCACTCTCAATCACTCTCAG
MUC1	TCTTTTCACATTTCAAACCTCCAG	TGAACTTAATATTGGAGAGGCCC
MX1	GCATCCCACCCTCTATTACTG	AGTCAATGAGGTCGATGCAG
MyD88	CCGCCTGTCTCTGTTCTTG	TTGTGTCTCCAGTTGCCG
RARRES1	GCTTCACTTCTTCAACTTCCG	GTAAAGACTCTGGGTTGTAGCG
STAT1	TGCTCCTTTGGTTGAATCCC	GGTACTGTCTGATTTCCATGGG
STAT2	ACCCTAATCAGAGCCCAAATG	TCAATCCAGACAGCCAAGTAC
TLR3	AAGGAAAGGCTAGCAGTCATC	GCAACTTCATGGCTAACAGTG
TNFSF10	GATGACAGTTATTGGGACCCC	TGACGGAGTTGCCACTTG
IFNα	CCCATTTCAACCAGTCTAGCAG	TGTGGGTTTGAGGCAGATC
IFNβ	AGGATTCTGCATTACCTGAAGG	GGCTAGGAGATCTTCAGTTTCG
IFNγ	GCAGAGCCAAATTGTCTCCT	ATGCTCTTCGACCTCGAAAC

**Figure 8:**
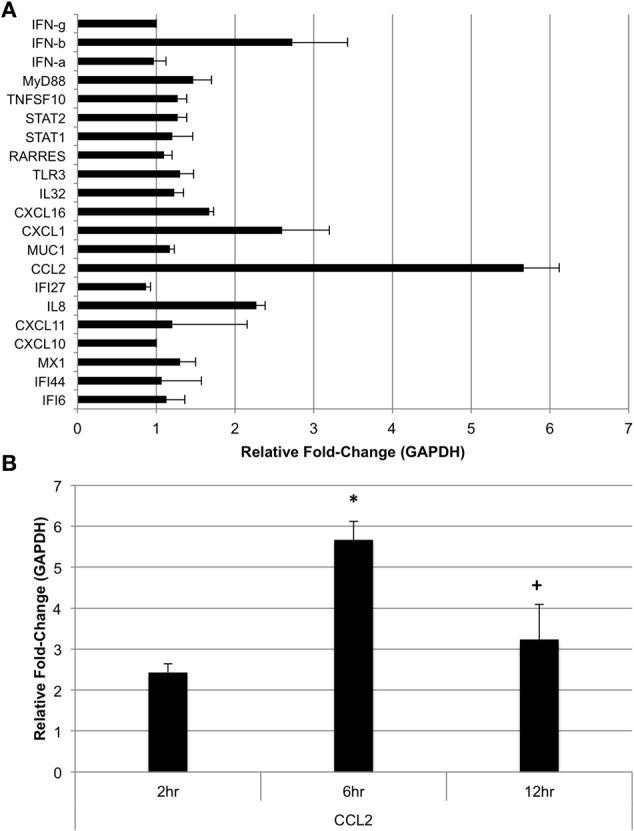
**Expression of selected genes in human bronchial epithelial cells post-*Alternaria alternata* spore challenge. (A)** BEAS-2B cells were challenged with 1 × 10^5^ *A. alternata* spores and cells were harvested for RNA extraction and qRT-PCR 6 h later. **(B)** CCL2 was assayed by qRT-PCR in cells harvested at 6 and 12 h post-spore challenge. ^*^p ≤ 0.01 vs. control; ^+^*p* ≤ 0.05 vs. 6 h.

The subset of genes chosen for microarray data validation via qRT-PCR at 24 h post-challenge with *A. alternata* were also assayed in human bronchial epithelial cells (BEAS-2B) 6 h post-challenge to determine if *A. alternata* spores drive expression of these genes at an earlier time point in this cell type. We also assayed for IFN-α, -β, and -γ at 6 h post-challenge to determine if early production of interferon might be responsible for the significant induction of interferon-inducible genes 24 h after challenge. The results are represented in Figure 8A. Interestingly, while the majority of the transcripts remain virtually unchanged vs. the control 6 h after spore challenge, four targets were up-regulated ≥2-fold. IFN-β, but not -α or -γ, is up-regulated ~2.7-fold vs. the control. IL8 and CXCL1, both neutrophil chemoattractants, are up-regulated 2.3- and 2.6-fold, respectively. The monocyte and basophil chemoattractant, CCL2, was the most strongly up-regulated of the gene subset 6 h after spore challenge at ~5.7-fold vs. the control, and appears to oscillate between ~3- and ~6-fold up to 24 h post-spore challenge (Figure 8B).

**Figure 9:**
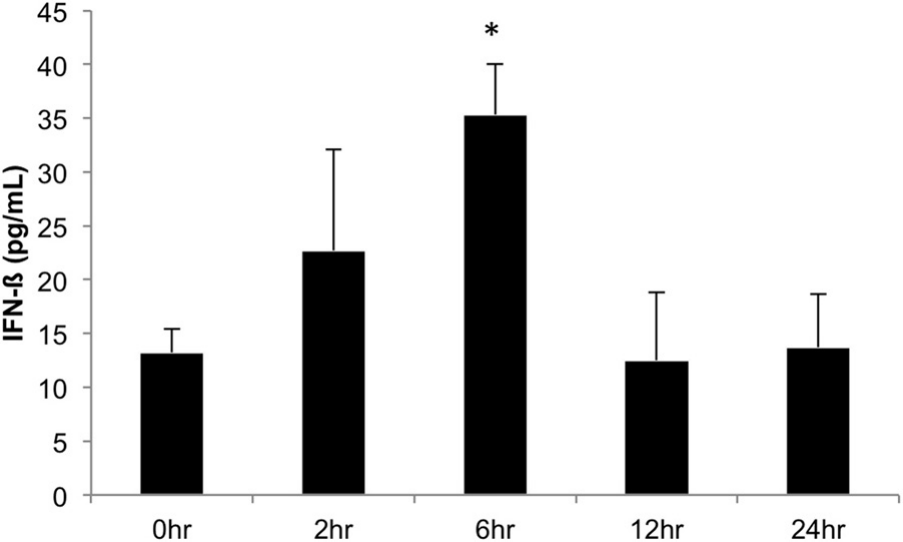
**IFN-β production in human bronchial epithelial cells over time in response to *Alternaria alternata* spores.** BEAS-2B human bronchial epithelial cells were challenge with 1 × 10^5^ *A. alternata* spores and INF-β levels were assayed by ELISA. ^*^p ≤ 0.002 vs. control.

**Figure 10:**
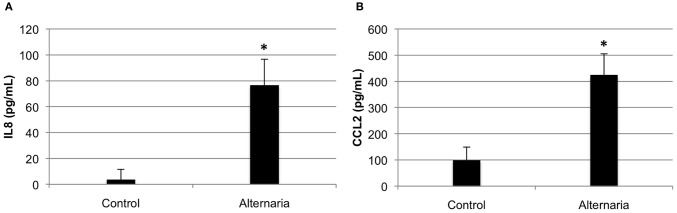
**Cytokine secretion by human bronchial epithelial cells in response to *Alternaria alternata* spores.** BEAS-2B human bronchial epithelial cells were challenged with 1 × 10^5^ *A. alternata* wild-type spores and ELISA was used to assay cell-free supernatants for **(A)** IL8 or **(B)** CCL2. ^*^p ≤ 0.01 vs. control.

We next investigated the ability of BEAS-2B human bronchial epithelial cells to secret IFN-β in response to challenge with *A. alternata* spores over time. Cells were challenged with 1 × 10^5^ spores and IFN-β levels were measured in the cell-free supernatant by ELISA. Figure 9 shows that IFN-β protein levels in the supernatant increase ~3-fold (*p* = 0.002) vs. the control 6 h post-challenge and return to basal levels by 12 h post-challenge. These results, combined with early induction of IFN-β gene expression, suggest that IFN-β might be a potential regulator of the significant up-regulation observed in interferon-inducible gene expression in human bronchial epithelial cells in response to *A. alternata* spore challenge. Finally, we investigated the ability of *A. alternata* spores to induce BEAS-2B cells to secrete IL-8 and CCL2 protein into supernatants. As expected based upon our gene expression results, we observed accumulation of these proinflammatory proteins following spore challenge compared to controls (Figure 10).

## Discussion

Exposure of lung epithelial cells with different stimuli (bacteria, virus, or fungus) can result in rapid, innate host defense responses. More specifically, epithelial cell activation can result in the release of proinflammatory cytokines and chemokines that attract inflammatory effector cells including neutrophils, macrophages, eosinophils, and lymphocytes. Recently, activation of epithelium and subsequent production of cytokines and chemokines have been of particular interest in allergic pathologies such as asthma and allergic rhinitis (Mills et al., 1999). Fungal spores are known to contain and/or secrete allergenic proteins, to be a source of immunogenic molecules including proteins and carbohydrates, and are associated with the development, onset, and exacerbation of allergic chronic respiratory diseases like asthma or rhinosinusitis (Hogaboam et al., 2005). Germinating Alternaria spores cannot enter or be taken up readily by epithelial cells or macrophages due to their large size (20–100 μm). Thus, the fungal cell surface and secreted products most likely play a major role in the Alternaria-human interaction. This is in contrast to spores of another respiratory disorder-associated fungus, Aspergillus, which can be internalized, survive, and germinate inside epithelial cells and also be taken up by resident and recruited effector cells like macrophages (Paris et al., 1997; Wasylnka and Moore, 2002; Han et al., 2011).

Our microarray and QRT-PCR experiments have provided for the first time a profile of the genes differentially expressed in a human cultured airway (bronchial) epithelial cell line following exposure to live Alternaria spores. Based upon our results, we found substantial evidence for the induction of many genes encoding cytokines and chemokines previously found to be associated with the innate immune response. For example, we found many chemokines in the set of genes that are specific to the cells exposed to *A. alternata*. Among these cytokines and chemokines, the interferon γ-inducible protein CXCL10 was previously found to be important in the recruitment of effector cells involved in host immune defense against intracellular pathogens (Luster et al., 1998; Dufour et al., 2002). The fact that CXCL10 was shown to be secreted by respiratory epithelial cells (Spurrell et al., 2005) and appears in our data set indicates it could be an important mediator of the epithelial cell response to Alternaria. A similar pattern was observed for CXCL11, a chemokine strongly induced by IFN-γ and IFN-β, and weakly induced by IFN-α. Other chemokines that appear to be induced by Alternaria are CXCL1 and CXCL6, potent neutrophil granulocyte chemoattractants that have been found in the early phase of experimental and clinical asthma and in the inflamed airway mucosa in COPD patients (Anderson, 2008). Interleukin-8 (IL8), a C-X-C chemokine, is a chemoattractant and activator for neutrophils and T-cells (Baggiolini et al., 1989; Thomas et al., 2000) and has been more recently shown to have a role in monocyte recruitment (Gerszten et al., 1999). As has been found with other fungi including Aspergillus, exposure to *A. alternata* induced high levels of IL-8 in the human respiratory epithelial cells in our experiments. The release of IL-8 into BEAS-2B lung epithelial cell cultures following exposure to Alternaria spores was confirmed by ELISA and supports our findings. In addition, the secretion of IL-8 and CXCL10 into primary normal healthy bronchial epithelial cell (NHBE) cultures after exposure to Alternaria spores was confirmed by ELISA (data not shown).

One of the more exciting observations we made was the up-regulation of Toll-like Receptor 3 (TLR3) in BEAS-2B cells treated with spores. Previously it was shown that TLR3-mediated challenge of airway epithelium by synthetic dsRNA induces multiple inflammatory genes including CCL2 and CXCL10, underscoring a shared role in the response to microbial stimuli (Takeda and Akira, 2004; Guillot et al., 2005; Ritter et al., 2005; Matsukura et al., 2006). It has been shown that viral infections trigger the activation of innate immune pathways and type I interferon response via TLR3 (Groskreutz et al., 2006; Iwamura and Nakayama, 2008). For example, it was previously reported that Rhinoviruses, a major agent responsible for the common cold, could increase the expression of TLR3 in human bronchial epithelial cells, and trigger exacerbation of the pulmonary allergic reaction through TLR3/TRIF-dependent or TLR3/IRF3-dependent pathways implying that TLR3 may play an important role in viral associated asthma (Hewson et al., 2005; Wang et al., 2009; Torres et al., 2010). In a different study, long-term activation of TLR3 induced inflammation and impaired lung function in mice (Stowell et al., 2009). In a recent paper (Bjornsdottir et al., 2011) it was shown that regulation of TLR3 is connected with asthma exacerbations. Down-regulation of TLR3 expression using siRNAs in rats caused a decrease of serum IgE levels and a reduction of IL4 mRNA expression in immune organs in an allergic asthma model (Meng et al., 2011). The same study suggested that TLR3 systemically modulated disease development in a rat asthma model by reducing serum IgE release via IL4 down-regulation, which may provide a vital clue for further research in asthma pathogenesis and suggest a new target for asthma treatment (Meng et al., 2011). It is important to note that in this study (Meng et al., 2011), mice were sensitized to and subsequently challenged with OVA (ovalbumin) and thus the role of TLR3 in regards to fungal induced allergic asthma has never been studied in any great detail.

Reports have also shown that respiratory epithelial cells produced interferon-β via TLR3 signaling in response to the invasive infection and allergy associated fungus, *Aspergillus fumigatus* (Beisswenger et al., 2012). In the same study the expression of CXCL10 paralleled TLR3 expression, suggesting that TLR3 is essential for CXCL10 release in respiratory epithelial cells (Beisswenger et al., 2012). Not only did we observe TLR3 up-regulated in our data set but we also found CXCL10 up-regulated substantially and confirmed by qRT-PCR (~80 fold change). In general, our microarray and qRT-PCR results suggest that it may be worth investigating the role of TLR3 in Alternaria-induced inflammation in the future.

As mentioned above, the classical ligand for TLR3 is double stranded RNA (dsRNA) from viruses. However, recent reports have shown that dsRNA may be derived from cells undergoing apoptosis or necrotic cell death (Yu et al., 2011). We found some evidence of induction of cell death associated pathways in our datasets. For example, TNFRSF21 may be involved in inflammation and immune regulation but is primarily known as a death receptor (Eder, 1997). In our data set this receptor appears down-regulated which would agree with our observation of no changes in epithelial cell morphology to indicate substantial cell death. TNFSF15 and TNFSF13B are genes that play an important role in immunity by T-helper cell activation (Eder, 1997), are known as death receptor ligands, and appear to be up-regulated in our dataset. Collectively, one might speculate that due to upregulation of TNFSF15 and TNFSF13B, TNFRSF21 may be down-regulated to reduce the overall amount of cell death. This might be expected when cells are exposed to cell death-causing agents like necrotrophic fungi such as Alternaria known to secrete toxins that induce apoptosis. Thus, up-regulation of TLR3 and associated signaling pathways may be due to release of RNA populations from dying cells that harbor secondary structures like hairpins or other small dsRNAs and may be the focus of future research in our laboratory. However, the amount of cell death observed in our studies was only 1–3% as revealed by LDH assays and thus the induction of interferon-associated genes via TLR3 signaling may not be due to host dsRNA. Moreover, MyD88 was significantly up-regulated in our data set. MyD88 has been found to activate several TLRs except TLR3 but functions as a negative regulator of TLR3 (Matikainen et al., 1996; Meylan et al., 2006; Maret et al., 2007). Thus, MyD88 may be essential in restricting TLR3 signaling, thereby protecting the host from unwanted immunopathologies associated with the excessive production of IFN-β (Siednienko et al., 2011). Indeed, our experiments revealed early induction of IFN-β both at the mRNA and protein levels in BEAS-2B exposed to Alternaria spores.

Retinoic acid signaling has been reported to be associated with response to viruses (Bowie and Fitzgerald, 2007) and its activation may further modulate transcription factors NFKB and IRF3/7 which induce the antiviral response (Kawai et al., 2005; Meylan et al., 2005, 2006). In our study we found many of the genes involved in the retinoic acid-response pathway to be up-regulated suggesting that retinoic acid might play a role in the response of epithelial cells following exposure to *A. alternata* spores. Indeed retinoic acid not only has been shown to play a role in cell death in myeloid cells by activating Interferon Regulatory Factor 1 and, implicitly, increasing interferon responses (Matikainen et al., 1996) but also has an exacerbating effect on asthma in mouse models (Maret et al., 2007). Moreover, persistent interaction between retinoids and some of their receptors, which are overexpressed by the bronchial epithelium of individuals with severe asthma, may contribute to abnormal repair and lead to deleterious airway remodeling (Druilhe et al., 2008). It is not known whether or not Alternaria secretes retinoic acid or similar molecules although retinoic acid and related molecules have been reported to be secreted by fungi (Prado-Cabrero et al., 2007). The genes responsible for retinoid biosynthesis have been discovered recently in Fusarium and previously in Neurospora (Prado-Cabrero et al., 2007). Indeed, our recent survey of the *A. alternata* genome indicates it does harbor the highly conserved biosynthetic gene cluster for retinoids (Lawrence, unpublished data). It will be interesting in the future to determine if this fungus actively secretes retinoids and if they play a role in inducing the retinoic-acid associated interferon response.

In conclusion, our microarray analysis demonstrates the ability of *A. alternata* spores to induce selective activation of different innate immunological pathways in human airway epithelial cells *in vitro*. This activation likely leads to the coordinated regulation of multiple, functionally related chemokines, cytokines and related signaling pathways in airway epithelium. It is likely that *A. alternata* proteins and/or secreted metabolites act as a major inducer of epithelial inflammatory responses. We are well aware that this study represents only a small, albeit critical step forward in understanding the interaction of Alternaria and mammals. However, this study paves the way for more complex studies in the future utilizing animal models of allergic airway inflammation and human cells derived from patients suffering from respiratory disorders.

## Materials and methods

### Fungal strain and growth conditions

*Alternaria alternata* (ATCC 66981) was used in all experiments. The strain was propagated on PDA (0.4% potato starch, 2% dextrose, 1.5% agar) incubated at 25°C in the dark. Conidia were collected in PBS by gentle agitation and enumerated on a hemacytometer.

### Cell culture and treatment

The human bronchial epithelial cell line BEAS-2B (Ke et al., 1988) derived from human bronchial epithelium transformed by an adenovirus 12-SV40 Adeno 12 hybrid virus, was cultured in 75 cm^2^ tissue-culture flasks in RPMI:1640 medium (Hyclone) supplemented with 10% FBS (Atlanta Biologicals, Atlanta, GA), 100 U/ml penicillin, and 100 μg/ml streptomycin at 37°C and 5% CO_2_. The BEAS-2B cell line is commercially available (CRL-9609) from American Type Culture Collection, Manassas, VA. Once the cells reached 80% confluence they were seeded in six-well tissue culture dishes, at a concentration of 1 × 10^6^ cells per well and allowed to adhere overnight. Cells were then washed once using Dulbecco's Phosphate Buffered Saline (Ca^++^/Mg^++^ free) (DPBS; Hyclone) and serum-starved for 2 h in RPMI: 1640 medium without supplements. Cells were then washed twice more with DPBS and cultured in a final volume of 1.5 mL RPMI: 1640 without supplements. Cells were treated with 5 × 10^5^ *Alternaria alternata* spores or left untreated (control). Cells were incubated at 37°C and 5% CO_2_ for 24 h. Following treatment, the supernatants were collected and debris was removed by centrifugation before storing at −80°C. The cells were washed twice with DPBS before RNA was extracted.

### Cytotoxicity measurement

The BEAS-2B human airway epithelial cells were plated and stimulated as described above. At the indicated timepoints, 50 μ l of cell culture supernatant was aseptically removed and the level of lactate dehydrogenase (LDH) was determined using the CytoTox 96 Non-Radioactive Assay Kit (Promega). Positive and negative control cells were treated with either 100 μ l of 10% Triton X-100 (1% v/v final concentration) or PBS respectively. Samples were incubated with LDH substrate for 5 min and then the absorbance was read on a microplate reader at 490 nm.

### RNA extraction

Total RNA was purified via a hybrid protocol using TRIzol (Invitrogen, Carlsbad, CA) and the RNeasy Cleanup Kit (Qiagen, Valencia, CA). TRIzol was added directly to cells in culture and gently collected and lysed using a cell lifter. RNA extraction was carried out per manufacturer's instructions until addition of ethanol. Following this step, the preparation was added to the RNeasy Mini Spin Column (Qiagen) and purified per manufacturer instructions with on-column DNase digestion. RNA was eluted in 100 μ l of RNase-free water and purity and concentration was assessed with microanalysis (Agilent Bioanalyzer 2100). RNA samples were stored at −80°C until microarray analysis.

### Microarray analysis and data processing

Affymetrix® GeneChip Human Genome U133 Plus 2.0 Array (HG-U 133 Plus 2.0) containing 47,000 genes and variants which represents ~39,000 characterized human genes was used for gene expression analysis. Array hybridization and scanning were performed in the Virginia Bioinformatics Institute Core Laboratory Facility (Blacksburg, VA) according to Affimetrix (Santa Clara, CA) protocols. Affymetrix-produced cel files (6 files) were deposited into GEO database (GSE32893—Human bronchial epithelial cells exposed to *A. alternata* spores). A probe set-based gene expression data file was generated from quantified image files with the RMA method (Irizarry et al., 2003). This dataset was analyzed with packages from the BioConductor tool suite (http://www.bioconductor.org/), using R version 2.12 (http://www.r-project.org/) and was annotated with Unigene annotation from the February 2009 mapping version of the human genome. The comparison of treatment with control, by the simultaneous analysis of all six data files containing information about the intensity values of the individual probes and the CEL files, (three for the control and three for the treatment), revealed a data matrix of probe sets in which each value indicate the calculated log abundance of each gene probe under the treatment and the control conditions. Using RMA, background subtraction and normalization gene data summarization was performed. Differential expression analysis was performed using the linear modeling features of the Affy limma GUI package available in the R software (Smyth, 2004). In order to correct the *p*-values the positive False Discovery Rate (Storey and Tibshirani, 2003) multiple-testing adjustment was applied. All genes in a comparison of interest (treatment vs. control) with an adjusted *p*-value ≤ 0.05 were considered as statistically significant regardless of the fold difference in expression level. However, genes found to be differentially expressed at ≥2-fold were considered to be of particular interest in these studies.

### Pathways analysis and network building

Pathway analysis and network were generated with of IPA (Ingenuity Systems, http://www.ingenuity.com/index.html). An Excel file containing the names (gene symbols) of significant genes and the corresponding Log_2_ fold changes was uploaded to the IPA software package. Differentially expressed genes were analyzed with the Ingenuity Knowledge Base that contains information from scientific publications regarding direct and indirect relationship between genes and proteins. Each edge represents associations for proteins or genes based on the Ingenuity knowledgebase and each node represents a gene. These associations consist of binding, activation, and inhibition and are represented with solid lines for direct and dashed ones for indirect associations. The intensity of the node color indicates the degree of up- (red) or down- (green) regulation. Nodes are displayed using various shapes that represent the functional class of the gene product.

### Quantitative (q) RT-PCR

Total RNA was extracted as described above, and ribosomal RNA integrity was confirmed by gel electrophoresis and spectrophotometry after elution. First-strand cDNA synthesis was performed using 1 ug of total RNA and random primers with the Tetro cDNA Synthesis Kit (Bioline) following the company's protocol. Standard RT-PCR reactions were set up for the generated cDNA and RNA-only controls and amplified for a constitutively expressed housekeeping gene (GAPDH) to confirm cDNA quality and rule out DNA contamination in the RNA samples.

Quantitative RT-PCR (qRT-PCR) was performed on an iCycler (BioRad) in duplicate under the following conditions: the reaction mixture for each sample included 12.5 ul SYBR Green Supermix (BioRad), 450 nm each of the forward and reverse primer, 2 ul cDNA template, and 9.5 ul nuclease-free water. Thermocycler conditions followed a typical 2-step protocol with an initial denaturation of 95°C for 3 min followed by 40 cycles of 95°C for 15 s and 53°C for 30 s. A melting curve was then generated by incremental temperature ramping (0.5°C every 10 s) from 55 to 95°C. The threshold cycle (C_t_) at which the change in reporter dye crossed the software-designated baseline was then auto-calculated by the iCycle iQ Optical System Software (BioRad) version 3.1.

Relative quantification of each gene was determined based on the PFAFFL method (Pfaffl, 2001) in which the relative expression ratio of the target gene in the treatment sample is calculated by comparing the PCR efficiency (E) and crossing point (CP) of the target gene with the E and CP of a non-regulated reference gene (GAPDH).

### Conflict of interest statement

The authors declare that the research was conducted in the absence of any commercial or financial relationships that could be construed as a potential conflict of interest.
